# Optimization of gabapentin dosage in pediatric patients with renal impairment: a physiologically based pharmacokinetic modeling approach

**DOI:** 10.3389/fphar.2025.1669990

**Published:** 2025-09-26

**Authors:** Xiaoli Qin, Chaozhuang Shen, Zhimin Li, Yujie Yang

**Affiliations:** ^1^ Department of Pharmacy, Affiliated Hospital of Southwest Jiaotong University, The Third People’s Hospital of Chengdu, Chengdu, Sichuan, China; ^2^ Department of Clinical Pharmacy and Pharmacy Administration, West China School of Pharmacy, Sichuan University, Chengdu, Sichuan, China; ^3^ Department of Pharmacy, Sichuan Academy of Medical Sciences & Sichuan Provincial People’s Hospital, School of Medicine, University of Electronic Science and Technology of China, Chengdu, Sichuan, China

**Keywords:** gabapentin, pediatric, renal impairment, physiologically based pharmacokinetic, dose adjustment

## Abstract

**Background:**

Gabapentin (GAB) is an adjunctive antiepileptic drug widely used in pediatric patients. However, little is known about its pharmacokinetics in pediatric patients under 3 years old or with renal impairment (RI). To address this, we developed a physiologically based pharmacokinetic (PBPK) model for precise dosing guidance.

**Methods:**

A PBPK model for GAB was first developed in healthy adults using PK-Sim® and then extended to pediatric populations, accounting for age-related physiological changes. For RI simulations, reduced glomerular filtration and tubular secretion were incorporated based on adult RI models.

**Results:**

The PBPK model accurately predicted GAB exposure in adults and children after single and multiple administration (geometric mean fold error <2). Plasma concentrations and PK parameters were similar in children under 3 years old and those aged 3-12. In pediatric RI patients under 12 years old, AUC_0-_  ∞ increased to 2.09-, 3.30-, and 31.67-fold for mild, moderate, and severe RI, respectively, compared to healthy children. Dosing frequency was adjusted to bid (mild RI), qd (moderate RI), and qod (severe RI), with an additional 50% dose reduction for severe RI.

**Conclusion:**

PBPK models provide better guidance for GAB dosing in pediatric patients with varying RI degrees, laying a foundation for precision therapy. This study is a significant step in optimizing GAB treatment for this high-risk pediatric population.

## 1 Introduction

Gabapentin (GAB) is a gamma-aminobutyric acid analogue that has been available since December 1993 (Swearingen et al., 2018). GAB is excreted in its original form from the systemic circulation through the kidneys, and its renal clearance (CL) is directly proportional to the creatinine clearance rate (Ccr). The changes in renal function will affect GAB pharmacokinetics (PK). When renal function is impaired, glomerular filtration rate (GFR) and tubular secretion are generally reduced due to kidney damage, leading to decreased GAB clearance and increased drug exposure (Blum et al., 1994). Based on guidance from the US Food and Drug Administration (FDA), the dosing of GBA in adult patients should be adjusted with a Ccr <60 mL/min (FDA, 2019). These dosing recommendations are a reduction in the total daily dose and a prolonged dosing interval for GAB.

The pharmacologic management of epilepsy in children is quite complex in clinical practice. GAB is indicated for use as an adjunctive antiepileptic drug in the treatment of complex partial seizures, with or without secondary generalization, in patients over 12 years of age. It is also approved for use as adjunctive therapy in treating partial seizures in pediatric patients aged 3–12 years (Tallian et al., 2004), but the use of GAB in children under 3 years of age is limited. The increasing use of GAB in pediatric centers necessitates further characterization of its PK in pediatric patients to determine dosing and optimize treatment, especially in pediatric patients with renal impairment (RI). However, in children with RI, appropriate dosing has not been established, which raises the risk of treatment.

Pediatric doses are typically derived from adult doses. Extrapolating adult dosages to children is challenging because it involves considering the developmental changes and maturation processes that affect drug absorption, distribution, metabolism, and excretion (Schijvens et al., 2020). There is no basis for extrapolating the recommended dose for adults with RI to children with RI. As a unique population, clinical PK trials tend to exclude children with or without RI and do not specifically analyze PK differences. Physiologically based pharmacokinetic (PBPK) models can be utilized to forecast PK profiles in simulated special populations and assist in making informed decisions about dosing strategies (Grimstein et al., 2019). PBPK models precisely assess age-related changes in anatomy and physiology, detailing the size, composition, and blood flow of various tissues and organs. This is particularly useful for understanding the change in CL in pediatric patients with limited data. The application of small-molecule drugs for pediatric patients has been extensively researched using PBPK models (Guo et al., 2023). A PBPK model for GAB has not been built for pediatric patients of different ages and renal function.

Therefore, this study aimed to create a PBPK model of GAB. This PBPK model was used to predict the PK of GAB in pediatric patients of differing ages and varying degrees of renal function and to establish a simulations-based dosage regimen. This approach aims to support extrapolated dose recommendations for GAB in children aged 1 month to 3 years or with RI.

## 2 Materials and methods

### 2.1 PBPK modeling development workflow and computer software

#### 2.1.1 Workflow

**FIGURE 1:**
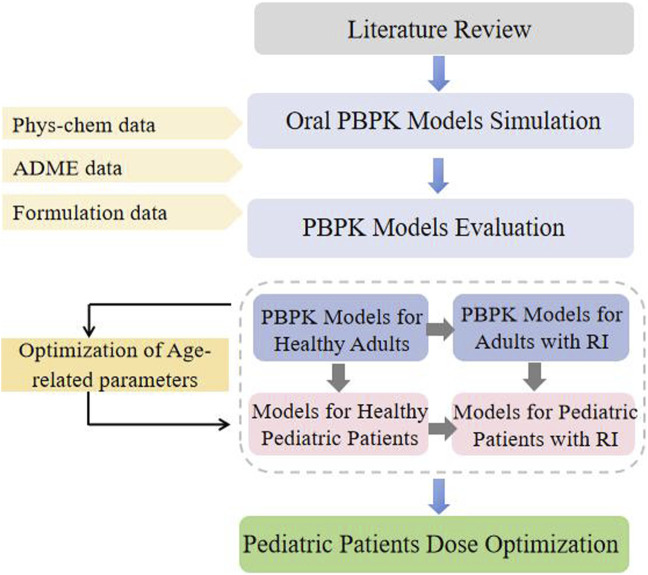
Schematic representation of the workflow of PBPK modeling development.

This study adopted a guidance-based workflow for PBPK modeling development in pediatric patients. A “top-down” strategy was used to facilitate modeling establishment (Figure 1). A PBPK modeling of GAB was developed and evaluated in healthy adults for p.o. Administration. The pediatric model is constructed by applying age-related changes in physiological parameters using the software’s built-in calculation methods. Afterward, the established healthy adults’ PBPK modeling of GAB was extrapolated to patients with RI by adjusting GFR, kidney size, and tubule secretion (TS_spec_). At this stage, GAB’s physicochemical and ADME properties remained the same. The simulated plasma exposure of virtual populations was compared with the observed concentration data to assess the accuracy of the PBPK modeling.

GAB’s physicochemical properties, ADME characteristics, and reported PK profiles following oral administration were obtained from literature searches in the DrugBank, Web of Science, PubMed, and Medline databases using the keywords “gabapentin” and “pharmacokinetics” (Table 1). The unique keywords for the pediatric patients were “pediatric,” “child,” “children,” “newborn,” “infant,” “adolescent,” or “teenager.” The collected literature was organized according to the dosing regimen and experimental data, and we excluded the study on determining GAB plasma concentration by the microbial method.

**TABLE 1:** Summary of input compound parameters of Gabapentin.

Parameters	Initial estimate	Final estimate	Source
Physico-chemical			
logP	0.52	−1.9–1.25	Drug bank
pKa-acid	4.63	4.63	ChemAxon
pKa-Basic	9.91	9.91	ChemAxon
MW, g/mol	171.2368	171.2368	Drug bank
Solubility at PH7, mg/mL	50	4.34–100	Drug bank
fup	0.73	0.91–0.67	Drug bank
Blood to plasma ratio (B/P)	0.97	0.97	Drug bank
ADME			
Partition coefficients	Schmitt		
Cellular permeabilities	PK-Sim® Standard		
CL_renal_ (mL/min/kg)	3.2		Parameter Identification
GFR filtration	1		Parameter Identification
Tubular secretion (TSspec 1/ min)	0.74		Parameter Identification

#### 2.1.2 Software

PBPK Modeling was conducted using the whole-body PBPK framework in the PK-Sim software (version 9.0, 2020, www.open-systems-pharmacology.org). Plasma concentration-time profile data were obtained from published literature using GetData Graph Digitizer (S. Fedorov, version 2.25.0.32). Some optimization outputs were manually adjusted for specific parameters to enhance the visual fit of the data. The model’s performance was verified through visual inspection of the predicted and observed plasma concentration-time curves and by comparing predicted PK parameter values with those observed in clinical PK studies.

### 2.2 Gabapentin modeling development

#### 2.2.1 PBPK model for healthy adults

We selected preexisting “healthy” populations and developed PBPK models for the oral administration of GAB in healthy adults. The represented GFR of healthy adults was 100 mL/min/1.73 m^2^. We used the Monte Carlo method to generate predicted values, and adopted a parameter identification method based on real-world observed data to estimate the renal clearance. We conducted single-dose simulations using GAB doses of 300 mg, 400 mg, and 600 mg. Additionally, we simulated multi-dose regimens for this population with the following doses: 600 mg and 1800 mg once daily (qd), 400 mg, 600 mg, 1200 mg, and 1600 mg three times daily (tid). We also included a regimen of 600 mg and a combination of 600 mg and 1200 mg taken twice daily (bid). The PBPK models for GAB were verified using clinical studies’ PK data.

#### 2.2.2 PBPK model for adults with RI

We utilized three levels of RI as recommended by FDA guidelines: mild, moderate, and severe. These categories were classified based on the GFR ranges: 30-59, 15-29, and <15 mL/min/1.73 m^2^. We created a virtual population of four adult patients with RI and set the representative GFR to 30, 15, and 5 mL/min/1.73 m^2^, respectively. We estimated the renal filtration rate using the method of fup*GFR, which is the product of the concentration of unbound drugs in plasma (fup) and the glomerular filtration rate. However, this model could not satisfactorily simulate the clearance rate of GAB, which only considers GFR. Therefore, we added parameters for renal tubular secretion. We conducted both single-dose and multi-dose simulations at various levels of renal function. The accuracy of the PBPK models was validated by comparing them with existing clinical data.

#### 2.2.3 PBPK model for pediatric patients with normal renal function

The PBPK models for healthy individuals with normal renal function were adjusted for age, height, and weight to create models suitable for pediatric patients with normal renal function. Age-related changes in physiological parameters were applied using the software’s built-in calculation methods. Based on a previous PK study, simulations were conducted for three age groups: children aged 1 month to less than 3 years, 3-12 years, and 12-17 years. Multi-dose simulations were performed for each age group, with simulated doses set at a single dose of 10 mg/kg and 5.7 mg/kg tid. The dosing regimens and demographics of pediatric patients with normal renal function are detailed in Table 2. Clinical observation data confirmed the accuracy of the models.

**TABLE 2:** Clinical pharmacokinetic reports used in Gabapentin PBPK modeling.

Source	Ethnic	Disease	Age (years) range	Weight (kg) M±SD	N	Proportion of male	Scheme	Dose
Adults								
Almeida et al. (2006)	European	Healthy	18–45	67.45 ± 9.24	24	0.48	Test capsule, po single dose	400 mg
							Reference capsule, po single dose	400 mg
Amini et al. (2010)	Asian	Healthy	18–62	—	12	—	Capsule, po single dose	400 mg
Backonja et al. (2011)	Caucasian	PHN	18–89	—	101	0.49	Capsule, Po tid, 7 days	600 mg
Chen et al. (2013)	American	Healthy	—	—	—	—	GR Tablet, Po single dose + high fat	600 mg
Cho et al. (2006)	Asian	Healthy	19–26	66.74 ± 8.31	26	1	Reference Capsule, Po single dose + high fat	400 mg
							Test Capsule, Po single dose	400 mg
Cowles et al. (2012)	Caucasian	MSVS	47–61	75 ± 19	27	0	ER Tablet, Po Pm,5 weeks	600 mg
							ER Tablet,po bid,5 weeks	600 + 1200 mg
	Caucasian	MSVS	49–61	78 ± 14	27	0	ER Tablet,Po bid,5 weeks	600 mg
							ER Tablet,Po bid,5 weeks	600 + 1800 mg
	Caucasian	MSVS	51–65	72 ± 13	27	0	ER Tablet,Po pm,5 weeks	1200 mg
							ER Tablet, Po bid,5 weeks	1200 + 1800 mg
Eckhardt et al. (2000)	European	Healthy	—	—	12	1.00	Capsule, Po single (Eldon et al., 1998) dose	600 mg
Eldon et al. (1998)	Caucasian	Healthy	18–50	≥50	11	—	Capsule, Po tid,7 days	400 mg
Gidal et al. (1996)	Caucasian	Healthy	26–38	72.3 + 12.95	10	0.50	Capsule, Po single dose	600 mg
Gidal et al. (1998a)	Caucasian	Healthy	22–49	74.29 ± 14	20	0.55	Capsule, Po tid, 3 days	400 mg
								600 mg
								1200 mg
								1600 mg
Gidal et al. (1998b)	American	Healthy	30–40	71.2 ± 14.8	9	0.33	Capsule, Po single dose	600 mg water
Gordi et al. (2008)	Caucasian	Healthy	18–65	77	24	0.46	ER tablet, Po qd,1 day	1800 mg
							ER tablet, Po bid, 1 day	600 + 1200 mg
							IR tablet, Po tid, 1 day	600 mg
							ER tablet, Po qd,7 days	1800 mg
							ER tablet, Po bid, 7 days	600 + 1200 mg
							IR tablet, Po tid, 7 days	600 mg
Jalalizadeh et al. (2007)	Asian	Healthy	25–32	77.4 ± 4.4	12	1	Tablet, Po single dose	400 mg
Kang et al. (2007)	Asian	Healthy	19–25	67.6 ± 7.05	30	1	Capsule, Po single dose	300 mg
KuKanich (2013)	Asian	Healthy	18–45	66.49 ± 6.59	24	1	Tablet, Po single dose	600 mg
Park et al. (2007)	Asian	Healthy	19–27	71.8 ± 6.7	24	1	Test Capsule, Po single dose	300 mg
							Reference Capsule, Po single dose	300 mg
Swearingen et al. (2018)	Caucasian	Healthy	29–55	—	14	0.50	IR tablet, Po tid, 5 days	600 mg
							GR tablet, Po qd, 5 days	1800 mg
Tjandrawinata et al. (2014)	Asian	Healthy	19–54	—	37	0.65	Test Capsule, Po single dose	300 mg
							Reference Capsule, Po single dose	300 mg
Toh et al. (2014)	Asian	Healthy	27–46	71.2 ± 10.6	10	1.00	Tablet, Po single dose	600 mg
Renal impairment adults								
Blum et al. (1994)	European	≥60 mL/min	18–75	≥50	60	—	Capsule, Po single dose	400 mg
		30–59 mL/min						
		<30 mL/min						
Lal et al. (2012)	Caucasian	15–59 mL/min	28–76	85.6	8	0.73	ER tablet, po single dose	600 mg
					1			
Pediatric patients								
Haig et al. (2001)	Caucasian	Healthy	1m-12 y	16–50	48	—	Capsule, Po single dose	10 mg/kg
Tallian et al. (2004)	Caucasian	partial seizures	3–14.5	39.1 ± 23.1	9	—	Capsule, po tid,1 day	5.7 mg/kg

PHN, post herpetic neuralgia; IR, Immediate-release; GR, gastro-retentive formulation; ER, gastric-retentive extended-release; MSVS, moderate to severe vasomotor sympto.

#### 2.2.4 PBPK model for pediatric patients with RI

Based on the PBPK models for adults with RI and children with normal renal function, we developed PBPK models for pediatric patients with varying degrees of RI: mild, moderate, and severe. To simulate drug exposure in this population, we selected a dosing regimen of 5 mg/kg administered tid, which corresponds to the recommended dosage range for healthy children as specified on the GAB label. The alterations in other physiological parameters attributable to RI in pediatric patients were assessed using the default values provided in PK-sim.

### 2.3 GAB modeling verification

Population simulations were conducted using PK-Sim to evaluate the predictive performance of the final models. This evaluation involved comparing the model outcomes with clinically observed data from other literature following various dosing regimens. The predicted and observed concentration-time profiles were compared through visual inspection, and the plasma drug concentration data fit was assessed. PK parameters of observed values were obtained from geometric or arithmetic means reported in the literature. If no PK parameters were reported, the intercepted plasma drug concentration values were used to fit by non-compartment analysis. The Equations 1, 2 were used to compare the differences between predicted and observed values of PK parameters C_max,_ t_max,_ area under the plasma concentration-time curve (AUC_0-_  ∞ ) to evaluate the accuracy of PBPK model. When the MFE and GMFE of all PK parameters were less than 2, we considered the PBPK model establishment successful.

$$MFE=\frac{1}{n}\times \frac{predicted}{observed}$$ (1)

$$GMFE=10^{\frac{1}{n}}\sum \log_{10}\frac{\text{predicted}}{\text{observed}}$$ (2)

According to conventional criteria, the model is considered acceptable if none of the predicted PK parameters exceed the corresponding observed value >2.0-fold. To further evaluate the model, we also conducted a (Equation 3) analysis for the GAB adult model using PK-Sim®. The sensitivity of parameters was calculated as follows.

$$\text{Sensitivity}=\frac{\Delta \text{PK}\;\text{parameter}}{\text{PK}\;\text{parameter}}\times \frac{p}{\Delta p}$$ (3)

An increase of 10% in the tested parameter (p) results in a corresponding 10% increase in the predicted PK parameter, according to a sensitivity value of +1.0.

### 2.4 Dose recommendations for pediatric patients

We used a PBPK model to establish the appropriate administration regimen for GAB in children under 3 years old. Next, we compared the drug exposure resulting from a 300 mg tid dose in adolescents over 12 years old. Finally, we evaluated potential dosing regimens for pediatric patients with different levels of renal function. Using the PBPK model, we simulated the recommended GAB dose for those with RI and compared their drug exposure to that of healthy children receiving a dose of 5 mg/kg tid. We predicted C_max_ and AUC values for various regimens in a simulation involving 1,000 subjects.

## 3 Results

### 3.1 PBPK modeling for healthy adult

**FIGURE 2:**
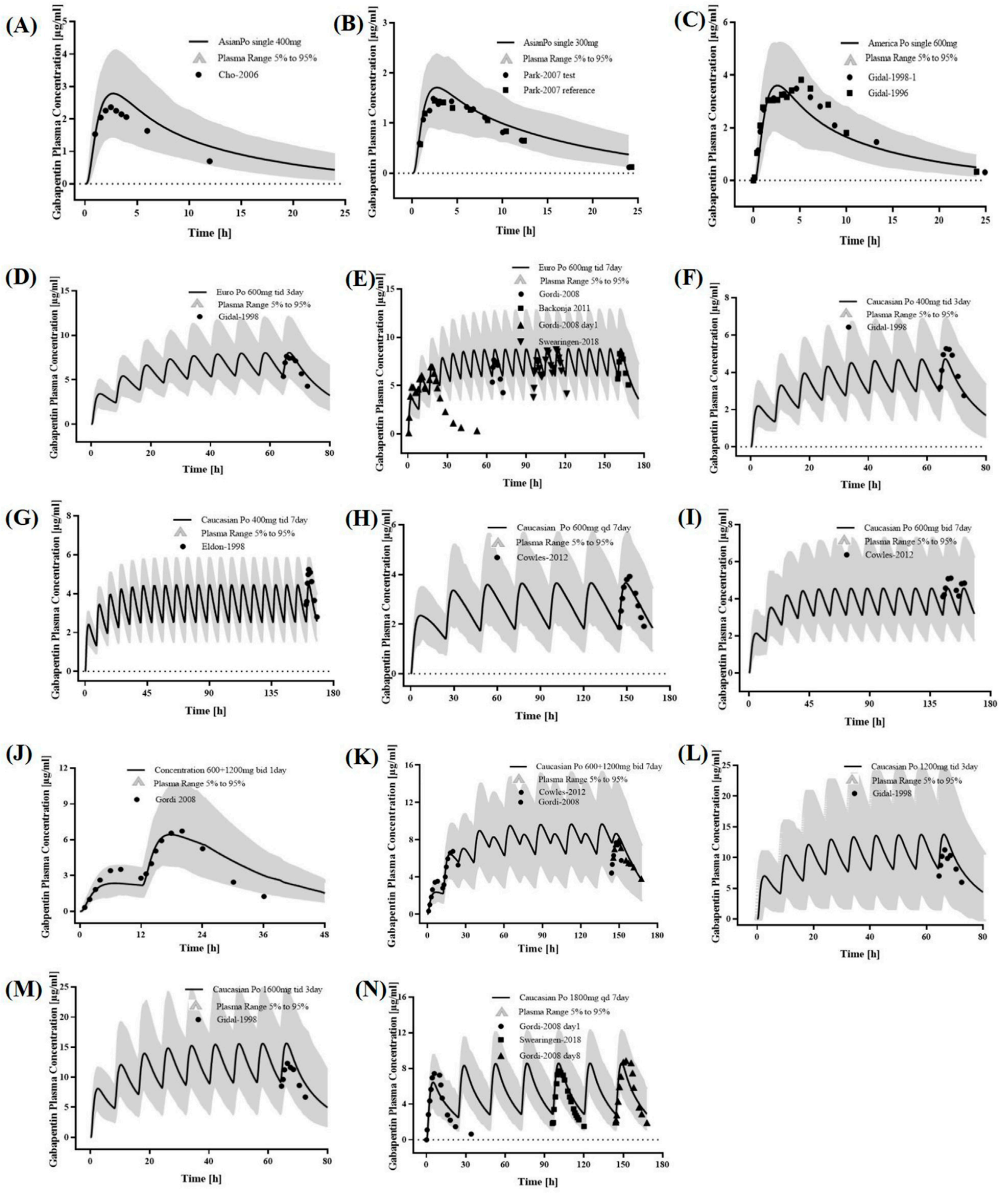
PK profiles in the healthy adult population. The po. administration at a single dose of **(A)** 400 mg, **(B)** 300 mg gabapentin in Asians; The po. administration at a single dose of **(C)** 600 mg gabapentin in White Americans; The po. administration at a dose of **(D)** 600 mg tid 3 days and **(E)** 600 mg tid 7 days gabapentin in Europeans; The po. administration at a dose of **(F)** 400 mg tid 3 days, **(G)** 400 mg tid 7 days, **(H)** 600 mg qd 7 days, **(I)** 600 mg bid 7 days, **(J)** 600 + 1,200 mg bid 1 day, **(K)** 600 + 1,200 mg bid 7 days, **(L)** 1,200 mg tid 3 days, **(M)** 1600 mg tid 3 days, and **(N)** 1800 mg qd 7 days gabapentin in caucasian. The observed concentration data were provided as the arithmetic mean values extracted from references. The solid line represented the predicted mean concentration, and the shaded area represented the predicted 5th to 95th percentile range.

We successfully constructed a PBPK model for healthy adults and validated it using clinical PK data. The established adult GAB PBPK model is shown in Supplementary Figure S1, which fits a single p.o well—administration at different doses of GAB on visual inspection. The GMFE of AUC_0-_  ∞ , C_max_, and t_max_ were 1.18, 1.11, and 1.31 in the established adult GAB PBPK model, respectively (Supplementary Table S1). For healthy adult PBPK model evaluation, 11 adult GAB PK studies following various dosing regimens were identified and used (Figure 2). The goodness of fit diagram of plasma drug concentrations showed that more than 96.80% of the predicted drug concentration values were within 2-fold error range of the observed values, about 90.40% and 77.60% of the predicted values were within 1.5-fold and 1.25-fold margin of error, respectively (Supplementary Figure S2). The MFE average and GMFE of all predicted PK parameters were within a 2-fold error range (Table 3).

**TABLE 3:** Predicted and observed PK parameters of Gabapentin in various dosing regimens.

Population	Dose	Method	AUC_0-_ ∞ (µg·h/mL)	C_max_ (µg/mL)	t_max_ (h)	Source
Healthy adults	Po single 400 mg	Predicted	33.99	3.08	2.50	Amini 2010
		Observed	26.18	3.41	3.02	
		MFE	1.30	0.90	0.83	
		Predicted	33.99	3.08	2.50	Jalalizadeh 2007
		Observed	29.09	3.33	3.13	
		MFE	1.17	0.92	0.80	
		Predicted	26.97	2.50	2.55	Almeida 2006 reference
		Observed	29.64	2.82	3.75	
		MFE	0.91	0.89	0.68	
		Predicted	26.97	2.50	2.55	Almeida 2006 test
		Observed	28.88	2.89	3.75	
		MFE	0.93	0.87	0.68	
		Predicted	26.97	2.50	2.55	Blum 1994
		Observed	38.95	2.91	3.15	
		MFE	0.69	0.86	0.81	
	Po single 600 mg	Predicted	50.98	4.62	2.55	KuKanich 2013
		Observed	48.71	4.85	3.17	
		MFE	1.05	0.95	0.80	
		Predicted	50.98	4.62	2.55	Toh 2014
		Observed	51.88	4.74	1.46	
		MFE	0.98	0.97	1.75	
	Po single 300 mg	Predicted	25.49	2.31	2.50	Kang 2007
		Observed	18.82	1.92	2.50	
		MFE	1.35	1.20	1.00	
	Healthy adults po. MFE		1.05	0.95	0.92	
	Healthy adults GMFE		1.18	1.11	1.31	
Adults evaluation	Po single 600 mg	Predicted	43.66	3.61	2.5	Gidal 1998-1
		Observed	41.79	3.98	4.02	
		MFE	1.04	0.91	0.62	
	Po single 300 mg	Predicted	43.66	3.61	2.5	Gidal 1996
		Observed	44	3.87	3.9	
		MFE	0.99	0.93	0.64	
		Predicted	27.24	1.73	2.94	Park 2007 test
		Observed	18.32	1.72	3	
		MFE	1.49	1.01	0.98	
		Predicted	27.24	1.73	2.94	Park 2007 reference
		Observed	17.72	1.71	2.50	
		MFE	1.54	1.01	1.08	
	Po single 400 mg	Predicted	37.46	2.83	2.82	Cho 2006
		Observed	22.51	2.7	2.5	
		MFE	1.66	1.05	1.13	
	Po 600 mg qd	Predicted	79.8	3.7	5.28	Cowles 2012
		Observed	62.929	5.004	7	
		MFE	1.27	0.74	0.75	
	Po 400 mg tid	Predicted	77.37	4.75	2.15	Gidal 1998
		Observed	138.32	5.26	2.49	
		MFE	0.56	0.90	0.86	
		Predicted	77.37	4.75	2.15	Eldon 1998
		Observed	208	5.24	2.03	
		MFE	0.37	0.90	1.06	
	Caucasian Po 600 + 1200 mg bid	Predicted	100.69	9.73	6.63	Cowles 2012
		Observed	141.301	8.92	6	
		MFE	0.71	1.09	1.11	
		Predicted	120.69	9.73	6.63	Gordi 2008
		Observed	144	6.997	6	
		MFE	0.84	1.40	1.11	
		Predicted	120.69	9.73	6.63	Gordi 2008
		Observed	144.6	6.537	6	
		MFE	0.83	1.49	1.11	
	Po 600 mg bid	Predicted	115.73	4.61	4.22	Cowles 2012
		Observed	107.84	6.229	8	
		MFE	1.07	0.74	0.53	
	Po 1200 mg tid	Predicted	208.49	13.91	2.14	Gidal 1998
		Observed	298.36	11.25	2	
		MFE	0.70	1.24	1.07	
	Po 1600 mg tid	Predicted	238.33	15.81	2.14	Gidal 1998
		Observed	355.12	12.30	2	
		MFE	0.67	1.29	1.07	
	Po 1800 mg qd	Predicted	185.91	6.64	5.94	Gordi 2008
		Observed	123	7.974	8	
		MFE	1.51	1.09	0.46	
		Predicted	185.91	8.7	5.94	Swearingen 2018
		Observed	105	8.43	7	
		MFE	1.91	1.03	0.53	
		Predicted	200.35	8.7	5.94	Gordi 2008
		Observed	133	9.585	8	
		MFE	1.51	0.91	0.46	
	Po 600 mg tid	Predicted	147.17	8.10	2.34	Gidal 1998
		Observed	225.57	7.65	2	
		MFE	0.65	1.06	1.17	
		Predicted	160.71	8.85	8	Gordi 2008
		Observed	141	8.166	8	
		MFE	1.14	1.08	1.00	
		Predicted	160.71	8.85	8	Backonja 2011
		Observed	166	9.07	2.31	
		MFE	0.97	0.98	3.46	
		Predicted	133.33	8.85	8	Gordi 2008
		Observed	136	7.455	14	
		MFE	0.98	1.19	0.57	
		Predicted	160.71	8.85	16	Swearingen 2018
		Observed	164	9.26	16	
		MFE	0.98	0.96	1.00	
	Adults evaluation po. MFE		1.06	1.03	1.03	
	Adults evaluation GMFE		1.34	1.15	1.28	
RI adults	Po single 600 mg (moderate RI)	Predicted	121.52	4.08	3.89	Lal 2012
		Observed	180	5.77	10	
		MFE	0.68	0.71	0.39	
	po singe 400 mg (mild RI)	Predicted	76.71	3.31	3.85	Blum 1994
		Observed	110	4.8	5.1	
		MFE	0.70	0.69	0.75	
	po singe 400 mg (severe RI)	Predicted	181.88	3.59	5.9	Blum 1994
		Observed	280	4.8	8.0	
		MFE	0.65	0.75	0.74	
	RI po. MFE		0.67	0.71	0.63	
	RI GMFE		1.48	1.40	1.67	
Pediatric Patients	3-12 years Po single 10 mg/kg	Predicted	44.55	4.05	2.46	Haig 2001
		Observed	36	4.52	2.52	
		MFE	1.24	0.90	0.98	
	3-15 years Po 5.7 mg/kg tid	Predicted	25.89	3.63	2.13	Tallian2004
		Observed	14.7	2.6	1.6	
		MFE	1.76	1.40	1.33	
	1m-3 y Po single 10 mg/kg	Predicted	40.69	4.34	2.17	Haig 2001
		Observed	25.6	3.74	2.14	
		MFE	1.59	1.60	1.01	
	Pediatric Patients po. MFE		1.53	1.15	1.11	
	Pediatric Patients GMFE		1.51	1.22	1.11	

^a^ tid, three times a day; bid, two times a day; qd, single daily dose.

### 3.2 PBPK modeling for adults with RI

**FIGURE 3:**
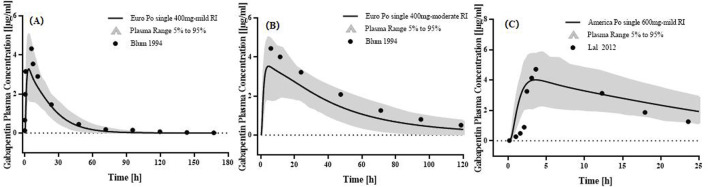
PK profiles in the adults with different levels of RI. The po. administration at a singe dose of **(A)** 400 mg gabapentin in Europeans with mild RI; The po. administration at a single dose of **(B)** 400 mg gabapentin in Europeans with moderate RI; The po. administration at a singe dose of **(C)** 600 mg gabapentin in White Americans with mild RI. The observed concentration data were provided as the arithmetic mean values extracted from references. The solid line represented the predicted mean concentration, and the shaded area represented the predicted 5th to 95th percentile range.

The PBPK model incorporated key physiological parameter changes associated with RI, including GFR, kidney size, and TS_spec_. As renal dysfunction intensifies, its related parameters decrease more (Supplementary Table S2). The plasma concentration predicted by the PBPK model for adults with RI aligned well with the observed values (Figure 3). The GMFE of PK parameters AUC_0-_  ∞ , C_max_, and t_max_ were 1.48, 1.40, and 1.67, respectively (Table 3). The goodness of fit diagram of plasma drug concentrations showed that more than 92.11% of the predicted drug concentration values were within 2-fold error range of the observed values, about 89.47% and 86.84% of the predicted values were within 1.5-fold and 1.25-fold margin of error, respectively (Supplementary Figure S3). The PBPK model predictions indicated that adults with RI experienced higher drug exposure compared to those with normal renal function. For a single dose, the AUC_0-_  ∞ increases to 2.05-fold, 3.51-fold, and 4.86-fold higher in mild RI, moderate RI, and severe RI, while there was less difference in C_max_. Sensitivity analysis (Supplementary Figure S6) indicated that fup was the most sensitive parameter affecting the peak concentration and systemic exposure of GAB (Absolute values of 0.81 and 0.82, respectively).

### 3.3 PBPK modeling for pediatric patients with or without RI

**FIGURE 4:**
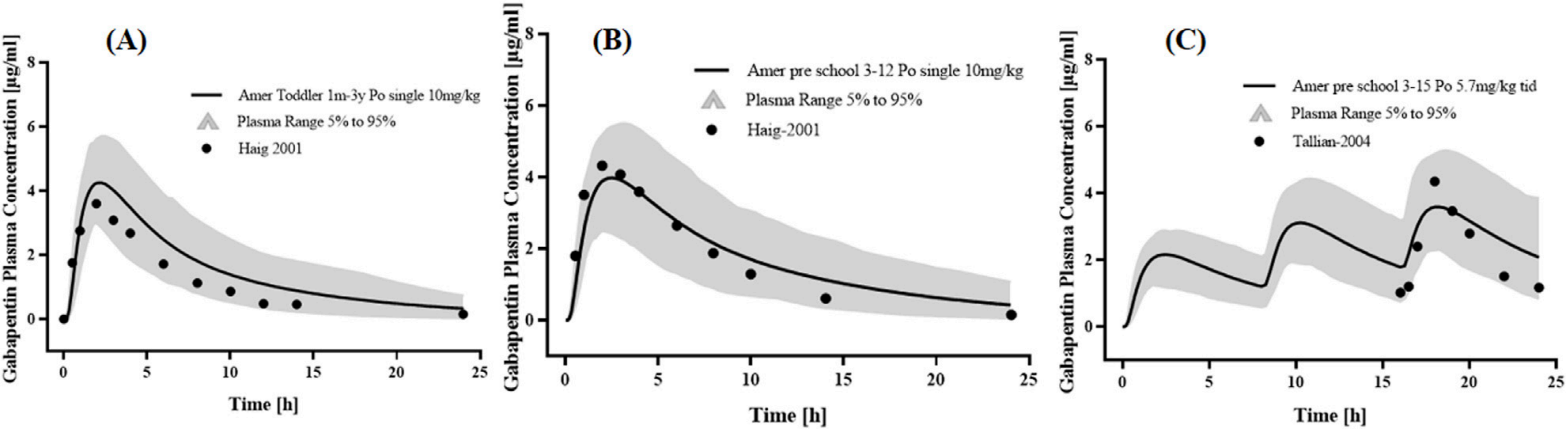
PK profiles in the pediatric patients with normal renal function. The po. administration at **(A)** a single dose of 10 mg/kg gabapentin in 1 month-3 years Americans; The po. administration at **(B)** a single dose of 10 mg/kg gabapentin in 3–12 years Americans; The po. administration at **(C)** a dose of 5.7 mg/kg tid gabapentin in 3–15 years Americans. The observed concentration data were provided as the arithmetic mean values extracted from references. The solid line represented the predicted mean concentration, and the shaded area represented the predicted 5th to 95th percentile range.

**FIGURE 5:**
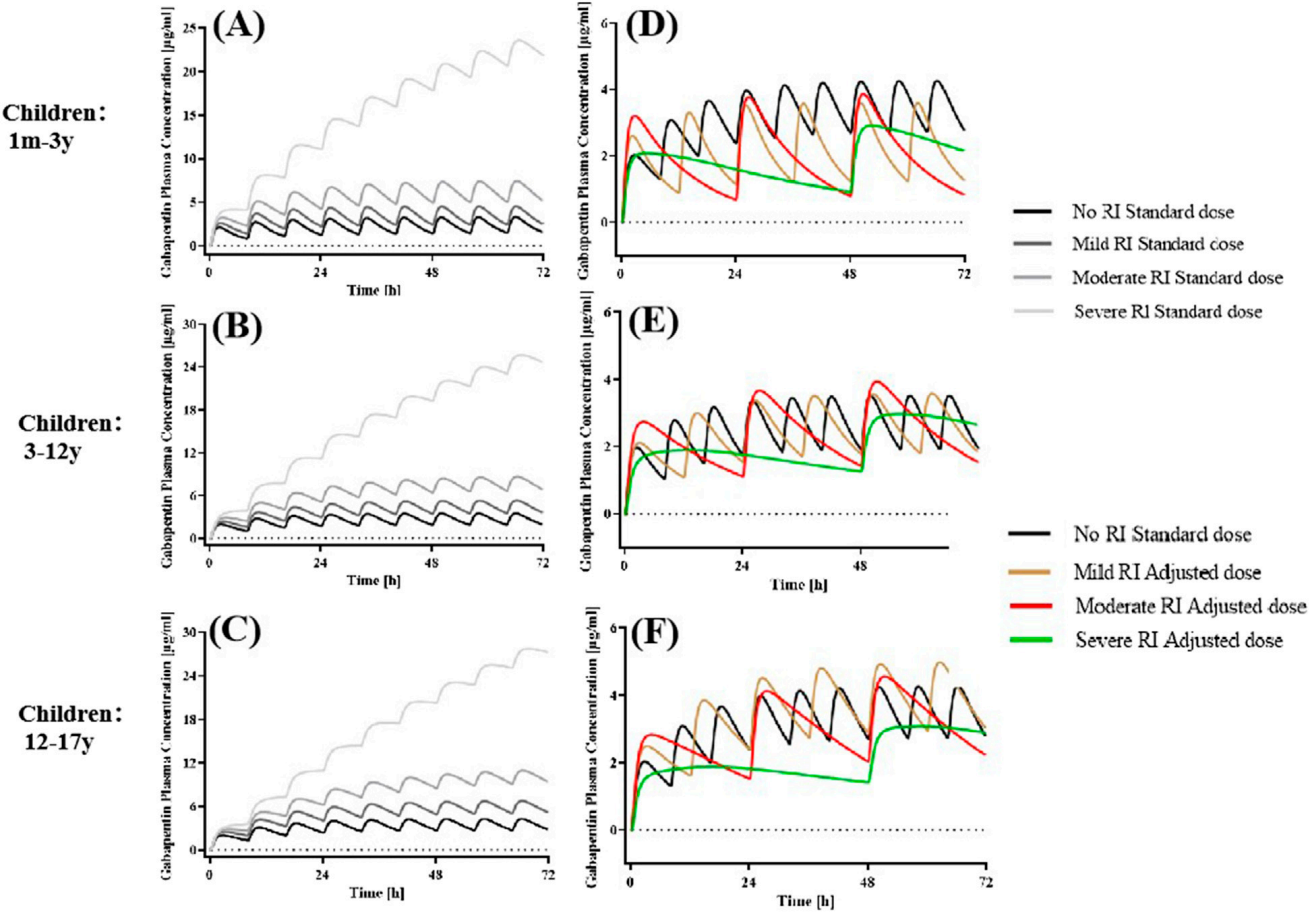
Simulated plasma concentration-time profiles in pediatric patients without RI and various degrees of RI receiving standard and adjusted dosages. The po. administration at a dose of 5 mg/kg tid GAB in **(A)** 1 m-3 y, **(B)** 3-12 years, and 300 mg tid in **(C)** 12-17 years with different degrees of RI. **(D–F)** Drug concentration-time profile of GAB following oral administration of 5 mg/kg (1m-12 y), 300 mg (12-17 years) bid in mild RI, 5 mg/kg (1 m-12 y), 300 mg (12-17 years) qd in moderate RI, and 2.5 mg/kg (1 m-12 y), 150 mg (12-17 years) qod in severe RI.

Two studies of p.o. The administration of GAB in Caucasians was used to optimize physiological parameters (Figure 4). The simulation results showed that the placement of age-related physiological parameters is suitable for GAB in pediatric patients. The GMFE of PK parameters AUC_0-_  ∞ , C_max_, and T_max_ in pediatric patients were 1.51, 1.22, and 1.11, respectively (Table 3). The goodness of fit diagram of plasma drug concentrations showed that more than 89.66% of the predicted drug concentration values were within the 2-fold error range of the observed values, and about 72.66% of the predicted values were within a 1.5-fold margin of error, respectively, by adjusting the physiological parameters related to RI (Supplementary Figure S4). Concerning adult PBPK models, we also developed a PBPK model for pediatric patients with RI. At the same dosage regimens (5 mg/kg tid), the AUC_0-_  ∞ values were 2.09-fold, 3.30-fold, and 31.67-fold in mild, moderate, and severe RI patients, respectively, compared to subjects with normal renal function under 12 years old (Figure 5). This indicated that dose adjustments were needed in pediatric patients with RI.

### 3.4 Dose recommendations for pediatric patients

**FIGURE 6:**
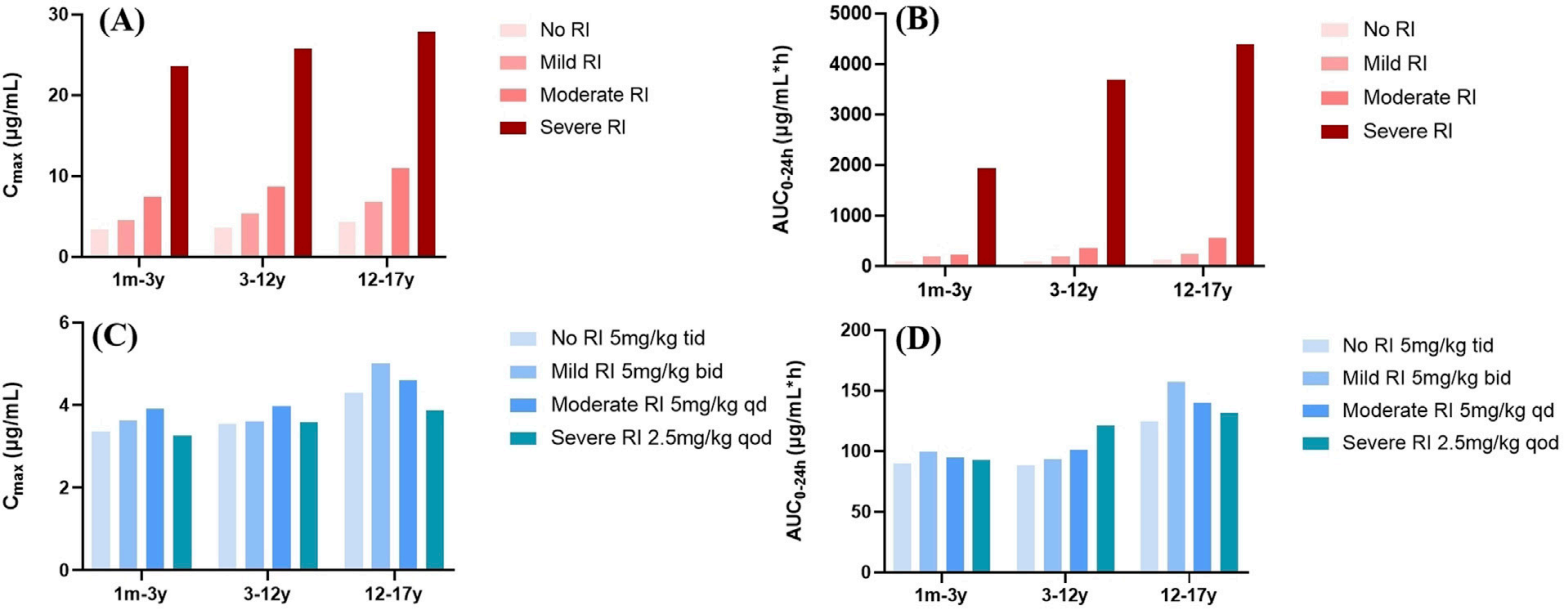
PK parameter profile of GAB in pediatric patients with different degrees of RI. **(A,B)** Mean C_max_ and AUC_0–24h_ profile of GAB following oral administration of 5 mg/kg (1m-12 y), 300 mg (12-17 years) tid in different RI. **(C,D)** Mean C_max_ and AUC_0–24h_ profile of GAB following oral administration of 5 mg/kg (1m-12 y), 300 mg (12-17 years) bid in mild RI, 5 mg/kg (1m-12 y), 300 mg (12-17 years) qd in moderate RI, and 2.5 mg/kg (1m-12 y), 150 mg (12-17 years) qod in severe RI.

The simulated plasma concentrations and PK parameters of GAB showed no significant differences between children below 3 years of age and those aged 3–12 years under identical dosing regimens (Figure 6). This suggests that a dosing regimen (5 mg/kg) based on body weight can be uniformly applied in pediatric patients up to 12 years of age. In addition, we categorized children into three groups according to international age classifications: young children (1 month–2 years), preschool children (2–6 years), and school-age children (6–12 years) (San and trock, 2017). The simulation results showed that the exact dosage was recommended for newborns and preschool children, consistent with previous research findings (Supplementary Figure S5).

Administration of the standard dose of GAB to pediatric virtual RI populations resulted in varying proportional increases in AUC_0–24h_ and C_max_ (Figure 6). Our findings demonstrated that GAB exposure was significantly higher in pediatric patients with moderate and severe RI compared to those with normal renal function. Consequently, based on the results of population simulations, we recommend a prolonged dosing interval for GAB in pediatric patients with RI to reduce the total daily dose. Specifically, for pediatric patients with mild, moderate, and severe RI, the frequency of administration was reduced to bid, qd, and qod, respectively. Moreover, the dose of pediatric patients with severe RI should also be halved (2.5 mg/kg). The results showed that PK parameters (AUC_0–24h_ and C_max_) of pediatric patients with RI were comparable after dose adjustment (Figure 6; Table 4).

**TABLE 4:** Gabapentin dosage based on renal function.

Renal function	Adults		Pediatric patients		
eGFR (mL/min/1.73 m^2^)	Total daily dose (mg/day)	Dose regimen (mg)	Adjust dose regimen		
			1 m-3 y	3-12 y	12-17 y
≥60	900	300 tid	5 mg/kg tid	5 mg/kg tid^#^	300 tid^#^
30–59	400	200 bid	5 mg/kg bid	5 mg/kg bid	300 bid
>15-29	200	200 qd	5 mg/kg qd	5 mg/kg qd	300 qd
15*	100	100 qd	2.5 mg/kg qod	2.5 mg/kg qod	150 qod

tid, Three times a day; bid, Twice a day; qd, Single daily dose; qod, every other day.

#, FDA, recommended dosage for children.

## 4 Discussion

The PBPK model has become an indispensable tool in drug development, especially in the study of children. Not only that, the application of the PBPK model in clinical practice is increasingly widespread, and its prediction of PK in special populations has also been widely accepted. Our PBPK models accurately predicted GAB exposures in adult and pediatric subjects with or without RI. The results of our study fill the blank of the use of GAB in pediatric patients with RI and have important significance for guiding clinical drug use.

GAB is used as adjuvant therapy for partial seizures (with or without secondary generalization). We have observed that the European Medicines Agency (EMA) currently approves GAB for children 6 years and older. The recommended starting dose is 10–15 mg/kg daily. The effective dose is typically between 25 and 35 mg/kg per day for children in this age group (EMA, 2021). While, the FDA approves GAB for use in children aged 3 years and older, with a starting dose that also ranges from 10 to 15 mg/kg per day and the effective dose ranging from 25 to 40 mg/kg per day, which (EMA, 2021). Several randomized controlled trials (RCTs) have established that GAB can be safely used in children under 3 years old (de Leeuw et al., 2019; Kaguelidou et al., 2019). However, there is currently no specific dosage recommendation available. Previous studies have suggested that drug exposure in older children when GAB is administered on a milligram per kilogram basis (Haig et al., 2001). Traditionally, pediatric dosages were often extrapolated from adult dosages based on age, weight, or body surface area. Applying PBPK modeling to determine initial pediatric clinical trial doses has gained increasing traction and regulatory acceptance in recent years (Heimbach et al., 2019; Johnson et al., 2021). Our PBPK modeling demonstrated that GAB plasma concentrations and PK parameters at standard doses are similar between children under 3 and those aged 3 to 12. This study established a GAB model for children under 3 years old, providing valuable evidence for pediatric practice.

GAB is a drug eliminated through glomerular filtration and tubular secretion (Beydoun et al., 1995). Human PK studies indicated that GAB is neither metabolized nor bound to serum proteins, and it is cleared solely through renal excretion (Blum et al., 1994)^.^ Consequently, GAB PK was susceptible to alterations caused by diminished renal function. Specifically, reduced renal function impairs GAB excretion, leading to its accumulation in the body (Zand et al., 2010). This suggests that dosage adjustments are necessary for individuals with RI (Randinitis et al., 2003; Ke et al., 2022). The FDA instructions of GAB explicitly state that GAB dosage should be adjusted based on CrCl in patients 12 years and older with RI. It is worth noting that in the period of RI, CrCl is affected by renal tubule secretion and overestimates GFR. Several studies have successfully applied PBPK modeling to simulate the PK of really excreted drugs in pediatric chronic kidney disease (CKD) populations (Yoon et al., 2019; Xu et al., 2022; Zhou et al., 2021). However, these investigations primarily focused on compounds without active tubular secretion, such as lamivudine and emtricitabine. Ye et al. utilized PBPK modeling to predict the PK of ertapenem in pediatric populations with CKD, considering glomerular filtration and tubular secretion (Ye et al., 2020). Our model incorporates GFR, kidney size, and TS_spec_ into the design of a representative individual (RI) population, which aligns with Ye’s considerations. This method assumes that a decline in glomerular filtration correlates with a reduction in tubular secretion, reflecting an overall loss of nephron function. Our study considered the advantages of renal tubular secretion, which is more comprehensive.

Interestingly, we found that the dose-adjustment protocol for RI patients in children was less consistent with that for adults, mainly the frequency in patients with severe RI. This difference arises because GAB drug exposure increases more substantially in children with severe RI than in adults. Children with severe RI showed a 31.67-fold increase in drug exposure compared to individuals with normal kidney function. The frequency and dose of severe RI in children should be further reduced compared to the dose-adjustment regimen of moderate RI. The core issue stems from the dynamic changes in children’s physiological parameters, resulting in a lack of pediatric-specific data. For example, the GFR of newborns is only 30% of that of adults, and it only approaches adult levels at the age of 2. When children have renal insufficiency, this immature glomerular function will be further damaged, leading to a significant decrease in drug clearance rate. In addition, there are differences in the distribution of body fluids, with children having a higher proportion of body fluids (75% in newborns compared to 60% in adults). The distribution volume of water-soluble drugs increases, but they are prone to accumulation when renal dysfunction occurs. Furthermore, children with low protein binding rates have lower plasma protein levels, especially in nephrotic syndrome, where the concentration of free drugs increases and the dosage needs to be adjusted more strictly (Edginton et al., 2006). Consequently, directly extrapolating dosage recommendations for adults with RI to children with RI is not advisable. PBPK modeling offers a robust scientific foundation for GAB dose adjustment strategies tailored to children with RI (Khalid et al., 2023), demonstrating its significant value in supporting medication decisions for pediatric populations (Freriksen et al., 2023).

While our study provides valuable insights into GAB dosing in pediatric RI patients, several limitations should be acknowledged. The model evaluation was constrained by the limited availability of pediatric data, with only three relevant studies identified through a comprehensive literature review. To support these findings, future validation through targeted clinical pharmacokinetic studies or therapeutic drug monitoring will be crucial to confirm the safety and efficacy of GFR-adjusted GAB regimens in children, particularly those under 3 years of age and RI patients. While developing this PBPK model represents a pivotal first step in pediatric dose determination for GBA therapy, the scientific approach must be complemented by concurrent evaluation of PK variations and age-related maturation of pharmacodynamic (PD) pathways that govern drug effects. Successful therapeutic optimization in pediatric populations necessitates a holistic assessment of PK/PD parameters, as the interplay between drug exposure dynamics and evolving biological responses fundamentally determines the risk-benefit profile. In addition, most GAB preparations on the market are tablets and capsules, often administered to children based on their body weight. However, these forms can be inconvenient for young patients. It is advisable to introduce more granules and solutions that would suit children better.

## 5 Conclusion

The PBPK models of GAB in adults were developed and adapted for young children, children, and adolescents with mild, moderate, and severe RI, incorporating age-related physiology, GFR, kidney size, and TS_spec_ adjustments and for pediatric patients with severe RI required careful consideration of both dosing frequency and amount. Our approach was particularly valuable when establishing clinical recommendations for drug administration of GAB in children.

## Data Availability

The original contributions presented in the study are included in the article/Supplementary Material, further inquiries can be directed to the corresponding author.

## Abbreviations

GAB, gabapentin; RI, renal impairment; PBPK, physiologically-based pharmacokinetic; CL, renal clearance; Ccr, creatinine clearance rate; PK, pharmacokinetics; PD, pharmacodynamic; GFR, glomerular filtration rate; FDA, US Food and Drug Administration; EMA, European Medicines Agency; qd, once daily; tid, three times daily; bid, twice daily; MFE, mean fold error; GMFE, geometric mean fold error; RCTs, randomized controlled trials; CKD, chronic kidney disease.
