# Supplementary material for: Optimization of gabapentin dosage in pediatric patients with renal impairment: a physiologically based pharmacokinetic modeling approach
Source: Front Pharmacol. 2025 Sep 26;16:1669990. doi: 10.3389/fphar.2025.1669990 (PMC12511071; doi:10.3389/fphar.2025.1669990)
Supplement: Supplementary file 1 [file Supplementaryfile1.docx]

Supplementary File

**Optimization of** **Gabapentin Dosage in Pediatric Patients with** **Renal Impairment: A Physiologically Based Pharmacokinetic Modelling Approach**

**Figure of contents**

**Figure S1** PBPK modeling for healthy adult

**Figure S2** Goodness-of-fit plot of Gabapentin in healthy adults

**Figure S3** Goodness-of-fit plot of Gabapentin in adults with different levels of renal impairment

**Figure S4** Goodness-of-fit plot of Gabapentin in pediatric patients with normal renal function

**Figure S5** Recommended dose adjustments for Gabapentin in pediatric patients (International Age Classifications)

**Figure S6** The sensitivity analysis for the Gabapentin adult model

**Table of contents**

**Table S1** Comparison of PBPK model for healthy adults predicted and observed PK parameters of Gabapentin

**Table S2** Optimization of Gabapentin Dosage in Renal Impairment Patients


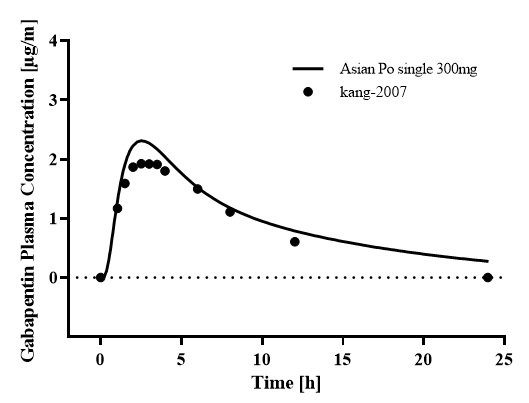

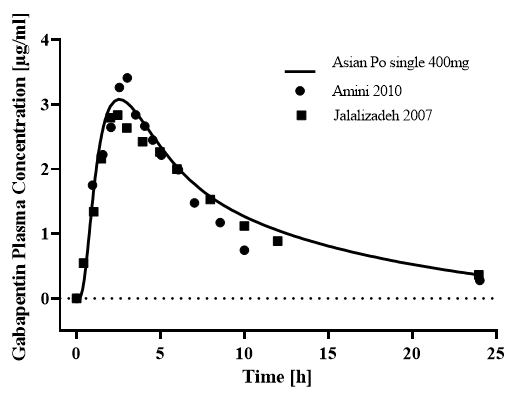

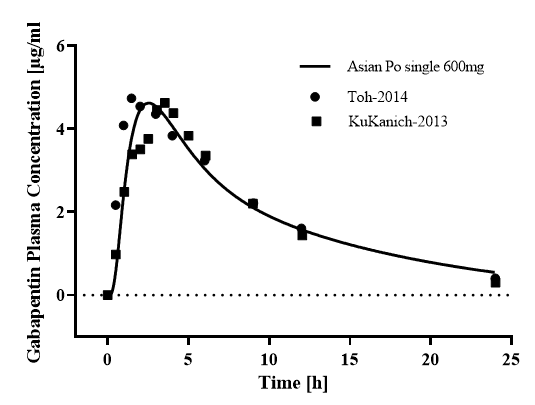

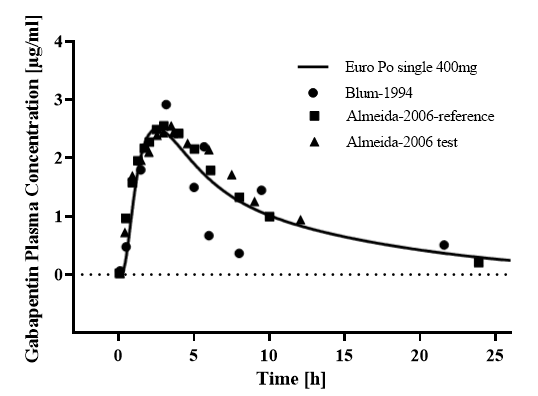

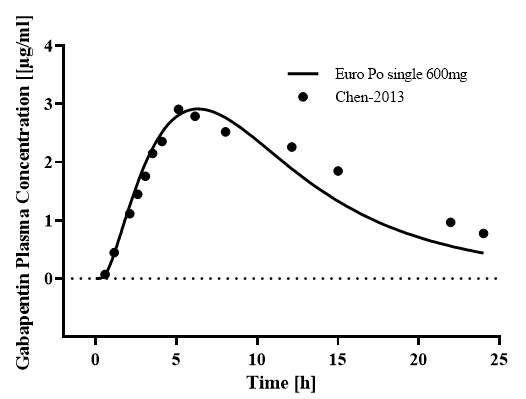


**(A)**

**(B)**

**(C)**

**(D)**

**(E)**

**Figure.S1 PBPK modeling for healthy adult.** Simulation (lines) of PK profles for a single po. Administration of **(A)** 300mg, **(B)** 400mg, and **(C)** 600mg gabapentin in a typical Asian individual and a single po. Administration of **(D)** 400mg and **(E)** 600mg gabapentin in a typical European individual. The observed concentration data were provided as the arithmetic mean values extracted from references.

**Figure.S2 Goodness-of-fit plot for (A)AUC, (B)C_max_, and (C)plasma concentrations of Gabapentin in healthy adults**. The solid line represented the line of identity, and the bold and dotted lines represented the 2-fold and 1.25-fold error range, respectively. The circles and squares indicated data for the PBPK modeling building and evaluation.

**（A）**

**（B）**

**（C）**

**Figure.S3** **Goodness-of-fit plot for (A)AUC, (B)C_max_, and(C) plasma concentrations of Gabapentin in adults with different levels of RI**. The solid line represented the line of identity, and the bold and dotted lines represented the 2-fold and 1.25-fold error range, respectively. The circles and squares indicated data for the PBPK modeling building and evaluation.


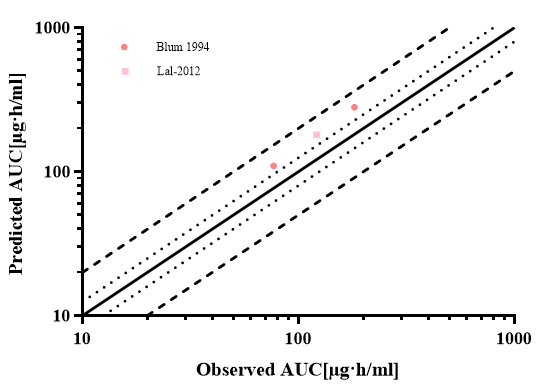

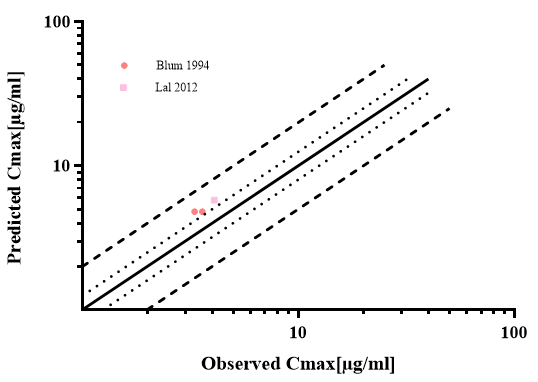

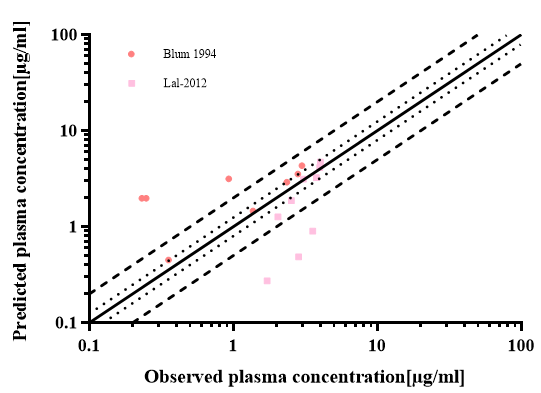


**(A)**

**(B)**

**(C)**


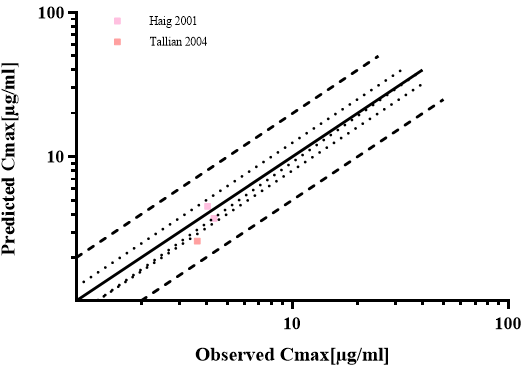

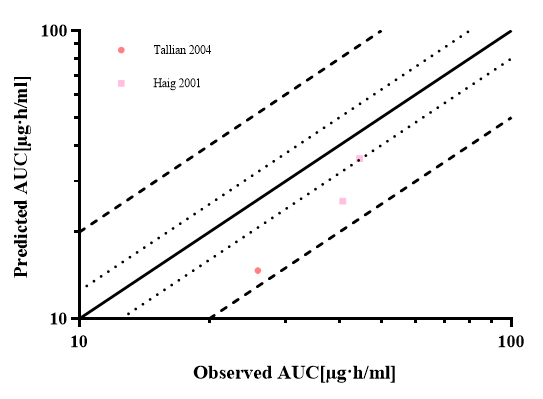

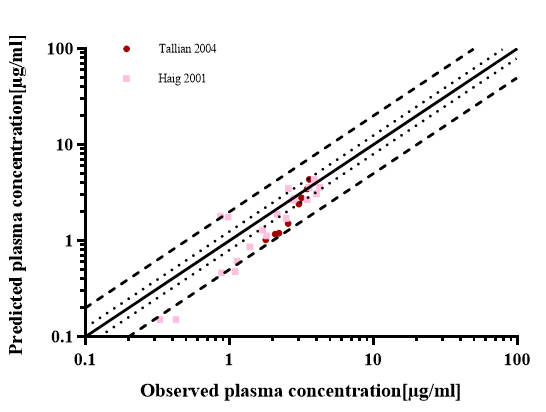


**（A）**

**（B）**

**（C）**

**Figure.S4 Goodness-of-fit plot for (A)AUC, (B)Cmax, and(C) plasma concentrations of Gabapentin in pediatric patients with normal renal function.** The solid line represented the line of identity, and the bold and dotted lines represented the twofold and 1.25-fold error range, respectively. The circles and squares indicated data for the PBPK modeling building and evaluation.


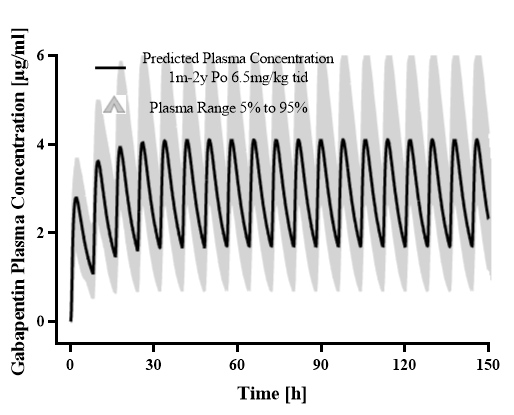

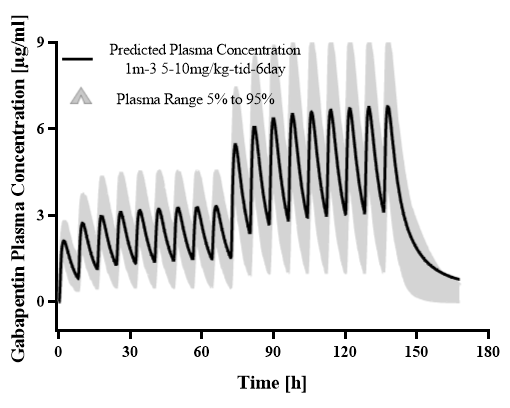


**（D）**


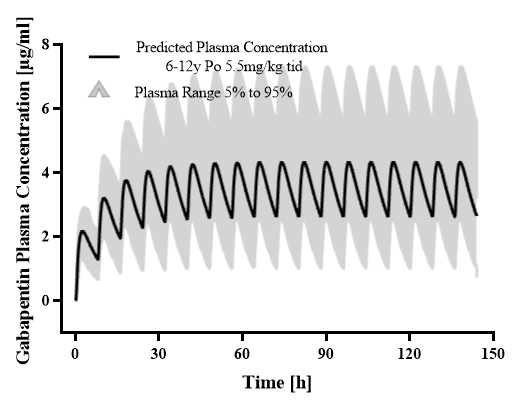

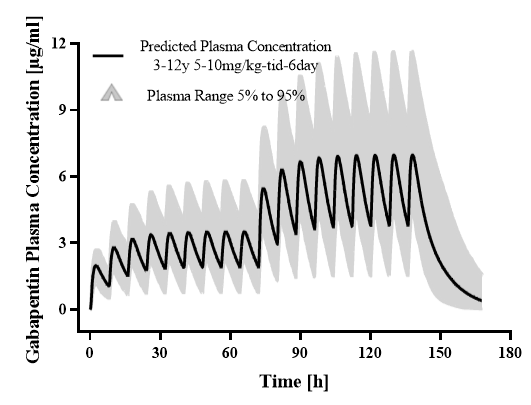

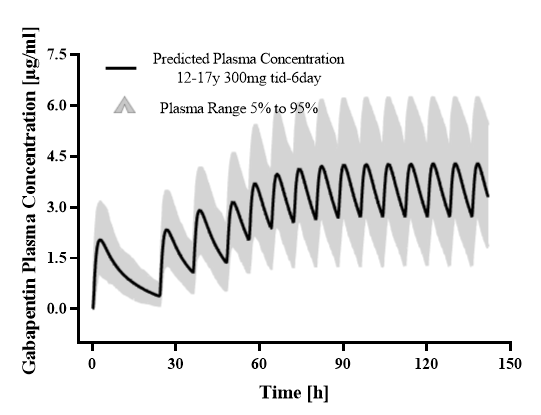


**（E）**

**（F）**

**（B）**

**（C）**

**(A)**

**Figure.S5 Recommended dose adjustments for Gabapentin in pediatric patients (International Age Classifications)**. The po. administration at a start-maintain dose of (**A**)5-10mg/kg tid gabapentin in 1m-3years Americans; The po. administration at a start-maintain dose of (**B**)10mg/kg tid Gabapentin in 3-12years Americans; and the po. Administration at a start-maintain dose of (**C**)300mg tid gabapentin 12-17years American adolescents. Recommended (**D**)5mg/kg tid adjustments for Gabapentin in neonates (1 month to 2 years), Recommended (**E**)6.5mg/kg tid adjustments for Gabapentin in preschool children (2 to 6 years), and recommended (**F**)5.5mg/kg tid adjustments for Gabapentin in school-age children (6 to 12 years).

**
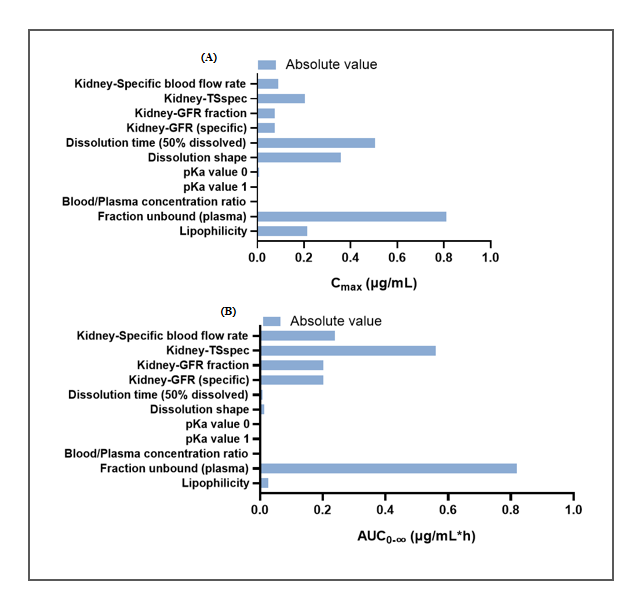
**

**Figure.S6 The sensitivity analysis for the Gabapentin adult model.** The sensitivity of the final model was measured by the relative change in a specific PK parameter following the administration of a single dose of 300mg gabapentin. A sensitivity value of +1.0 indicates that a 10 % increase in the tested parameter would result in a 10% increase in the PK parameter. The sensitivity values are presented in the figures as absolute values.

**Table. S1 Comparison PK parameters of Gabapentin PBPK Models in Healthy Adults**

| **Parameter** | **Predicted mean** | **Observed mean** | **MFE** | **GMFE** |
| --- | --- | --- | --- | --- |
| AUC_0-∞_ (µg·h/ml) | 34.54 | 34.02 | 1.05 | 1.18 |
| C_max_ (µg/ml) | 3.15 | 3.36 | 0.95 | 1.11 |
| t_max_ | 2.53 | 2.99 | 0.92 | 1.31 |

Note: AUC_0-∞_: 0 to infinity hours blood drug concentration-area under the time curve; C_max_: Peak blood concentration; t_max_: Peak concentration time; Mean fold error (MFE):$MFE=\frac{1}{n}\times\frac{\text{predicted}}{\text{observed}}$, Geometric mean fold error (GMFE):

**Table. S2 Optimization of Gabapentin Dosage in Renal Impairment Patients**

| Parameters | POPULATIONS | | | | | | | |
| --- | --- | --- | --- | --- | --- | --- | --- | --- |
|  | Adults | | | | Pediatric Patients | | | |
|  | No RI | Mild RI | Moderate RI | Severe RI | No RI | Mild RI | Moderate RI | Severe RI |
| GFR range(mL/min/1.73m^2^ ) | >60 | 30~59 | 15~29 | <15 | >60 | 30~59 | 15~29 | <15 |
| Represented GFR(mL/min/1.73m^2^ ) | 100 | 30 | 15 | 5 | 100 | 30 | 15 | 5 |
| Kidney size baseline (L) | 0.44 | 0.44 | 0.23 | 0.2 | 0.44 | 0.44 | 0.23 | 0.2 |
| TS_spec_ (1/min) | 0.74 | 0.44 | 0.24 | 0.04 | 0.74 | 0.44 | 0.24 | 0.04 |
| Maintenance dose | | | | | | | | |
|  | 300mg tid | 200mg bid | 200mg qd | 100mg qd | 5mg/kg tid | 5mg/kg bid | 5mg/kg qd | 2.5mg/kg qod |

Note: RI, Renal Impairment; GFR, glomerular filtration rate；TS, tubular secretion；tid = three times a day; bid = two times a day; qd = single daily dose; qod, every other day.

$CL_{sec}=\frac{QR*fub*CL_{int}}{QR+fub*CL_{int}};CL_{r}=\left( fub*GFR+\frac{QR*fub*CL_{int}}{QR+fub*CL_{int}} \right)^{*}(1-FR)$. Where fub = free drug fraction in blood, GFR= glomerular fltrationrate, OR = renal blood flow, CLint = endocrine activity of renal tu-bules. and FR= renal tubular reabsorption fraction.
